# Shedding of host autophagic proteins from the parasitophorous vacuolar membrane of *Plasmodium berghei*

**DOI:** 10.1038/s41598-017-02156-7

**Published:** 2017-05-19

**Authors:** Carolina Agop-Nersesian, Mariana De Niz, Livia Niklaus, Monica Prado, Nina Eickel, Volker T. Heussler

**Affiliations:** 1 Institute of Cell Biology, University of Bern, 3012 Bern, Switzerland; 2 Graduate School for Cellular and Biomedical Sciences, University of Bern, 3012 Bern, Switzerland; 3 Bernhard Nocht Institute of Tropical Medicine, 20359 Hamburg, Germany; 4 Department of Molecular and Cell Biology, Henry M. Goldman School of Dental Medicine, Boston University, MA, 02118 USA; 5 Wellcome Centre for Molecular Parasitology, University of Glasgow, G12 8QQ Glasgow, UK; 6 Centro de Investigación en Enfermedades Tropicales (CIET), Universidad de Costa Rica, San José, Costa Rica USA; 7 CSL Behring, Bern, Switzerland

## Abstract

The hepatic stage of the malaria parasite *Plasmodium* is accompanied by an autophagy-mediated host response directly targeting the parasitophorous vacuolar membrane (PVM) harbouring the parasite. Removal of the PVM-associated autophagic proteins such as ubiquitin, p62, and LC3 correlates with parasite survival. Yet, it is unclear how *Plasmodium* avoids the deleterious effects of selective autophagy. Here we show that parasites trap host autophagic factors in the tubovesicular network (TVN), an expansion of the PVM into the host cytoplasm. In proliferating parasites, PVM-associated LC3 becomes immediately redirected into the TVN, where it accumulates distally from the parasite’s replicative centre. Finally, the host factors are shed as vesicles into the host cytoplasm. This strategy may enable the parasite to balance the benefits of the enhanced host catabolic activity with the risk of being eliminated by the cell’s cytosolic immune defence.

## Introduction

The liver infection represents the first clonal expansion of the malaria-causing parasite *Plasmodium* in the mammalian host, ultimately leading to the symptomatic erythrocytic stage. *Plasmodium* burden in the liver can be controlled *in vivo* by interferon (IFN) signalling-mediated recruitment of innate immune cells^1^ and is complemented by hepatocyte-mediated intracellular defence mechanisms such as actin polymerization^2^, apoptosis^3, 4^ and selective autophagy^5–8^.

In *Plasmodium berghei*-infected hepatocytes, the host’s autophagy machinery has an ambivalent impact on parasite survival. The metabolic function of canonical autophagy supports *Plasmodium* differentiation and proliferation into numerous progeny merozoites^7, 9^. Thus, a constitutively active autophagy pathway appears pivotal for nutrient scavenging of the parasite in the liver. However, the membrane-enclosed replicative niche of *Plasmodium*, the so-called parasitophorous vacuolar membrane (PVM), becomes recognized by the hepatocyte’s autophagic machinery causing the elimination of the parasites through the autodegradative pathway^7, 8^. Molecular components associated with the PVM are ubiquitin, sequestosome1 (SQSTM1/p62) and the autophagy-related protein 8 (Atg8/LC3)^7, 9^, which are features of a rather selective type of autophagy^10^. In *Plasmodium* liver stages, LC3 becomes directly incorporated into the PVM depending on the lipidation machinery, which are components of the ubiquitin-like conjugation system^7–9, 11^. Especially this core molecular machinery is shared between the different autophagic pathways^12^.

To escape the host’s autodigestive process, intracellular pathogens have evolved various strategies. Intracellular bacteria that reside only transiently in their vacuolar compartments, such as *Listeria monocytogenes* and *Shigella*, mainly circumvent their recognition to prevent secondary autophagocytosis. After exposure to the host cytoplasm these bacteria either utilize actin-comet motility to shed the recruited autophagy proteins or secrete a bacterial effector to shield autophagy targets on the surface, respectively^13, 14^. However, bacteria that persist in their vacuolar niche often inhibit the downstream autophagy machinery. *Coxiella burnetii* can only persist in the autophagosome-like compartment by delaying lysosome fusion until developing into an acid-tolerant stage^15, 16^. In contrast, many viruses such as *Herpes simplex* interfere with the host signalling pathway to block initiation of autophagy^17^. In the *Plasmodium*-related parasite *Toxoplasma gondii*, autophagy-mediated immunity results in the disintegration of the PVM and therefore clearance of the infection^18, 19^.

Yet, it is unclear how *Plasmodium* circumvents the host’s autodegradative pathway without restricting the salvage of the autophagy-derived metabolites. As an adaptation to their hepatocyte environment, *Plasmodium* remodels the nascent PVM. Besides compositional changes by expression and export of various PVM-associated proteins^20–26^, the PVM additionally undergoes morphological changes, such as the formation of the tubovesicular network (TVN)^11^. This exomembranous system further expands the PVM into the host cytoplasm.

By taking advantage of a wide spectrum of imaging techniques^27^, we investigated the role of the TVN in controlling host autophagic proteins during liver stage development. Based on our quantitative fluorescent microscopy techniques we captured spatial and temporal changes of the PVM-associated host autophagy proteins *in vivo* and *in vitro*. Our high-resolution multi-dimensional imaging resolved the morphological details of the PVM and TVN architecture. Interestingly, the compartmentalisation of this exomembranous system appears to isolate host effector proteins from the PVM, representing an auxiliary function of the TVN against the hepatocyte’s cytosolic immune mechanism. Our study suggests a novel mechanism to control the load of host-autophagic protein on the surface of the PVM.

## Results

### Progressive removal of PVM-associated LC3 into the tubovesicular network

**Figure 1:**
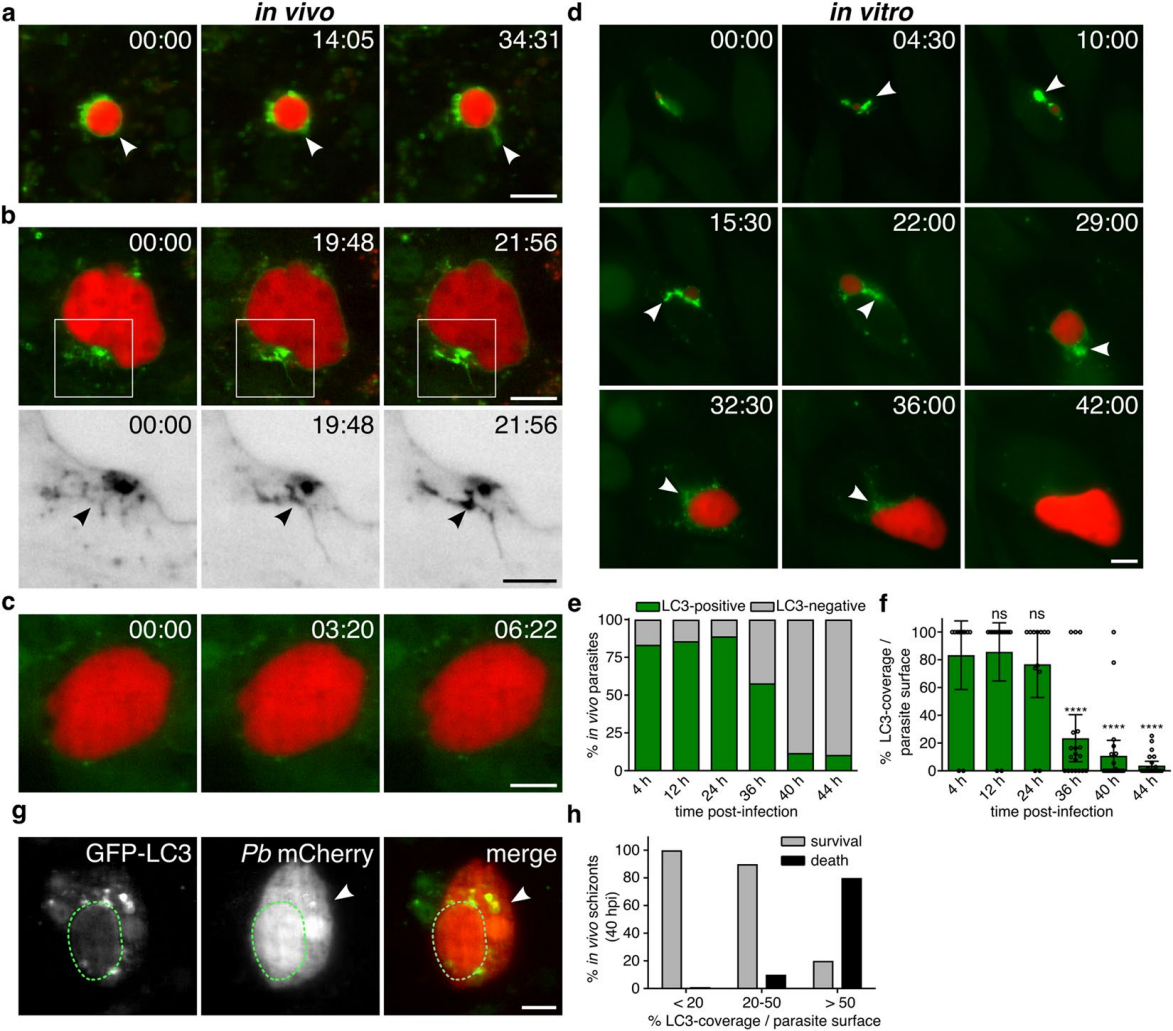
Comparison of the PVM–associated LC3 clearance during liver stage development *in vivo* and *in vitro*. **(a–c)** Liver intravital time-lapse of *gfp-lc3* transgenic mice infected with schizonts of different developmental stages. **(a)** Young *Pb*mCherry schizont 25 hpi (red) shows LC3 (green) incorporated into the PVM and the dynamic TVN (white arrowhead). Movie interval 2 min. **(b)** Proliferating liver schizonts 30 hpi accumulating LC3 in the evolving TVN. Lower panel shows magnification of the depicted inset. Arrowhead highlights increasing GFP-LC3 signal in the TVN. Acquisition interval 5 s. **(c)** Mature *Pb*mCherry schizont 44 hpi (red) entirely cleared from PVM-associated LC3 (green). Movie interval 2 s. **(d)** Wide field *in vitro* time-lapse microscopy of GFP-LC3 expressing HeLa cell (green) infected with mCherry expressing parasites (red). Long-term movie of the *P. berghei* liver stage showing the accumulation of GFP-LC3 in the evolving TVN (white arrowhead) and a progressive loss from the PVM. With completion of parasite division, LC3 is completely lost from the PVM. Imaging interval 30 min. **(e–h)** *In vivo* quantification of the LC3 accumulation at the PVM at indicated times of the liver stage development. **(e)** Stacked column graph of the overall percentage of LC3-positive and -negative parasites. **(f)** LC3-coverage of the PVM normalized to parasite surface area. **(g)** Still image of a liver intravital movie showing the elimination of mature schizonts. Green area outlines the PVM. The necrotic schizont shows release of parasite mCherry into the host cytoplasm (white arrowhead) **(h)** Quantification of the parasite survival rate in correlation to the LC3-load measured at the PVM. Time stamps, min:s **(a–c)** and h:min **(d)**. Scale bars, 10 µm.

The autophagy-mediated host defence contributes to the restriction of the pre-erythrocytic *P. berghei* development^7, 8^. Less than 50% of the infectious sporozoites successfully develop *in vitro* into mature schizonts^7^. In order to survive, proliferating parasites might circumvent the host’s autophagic surveillance pathway. We used exogenously expressed GFP-LC3 as a key representative marker for autophagy to characterise the parasite’s interaction with the host autophagic machinery. To follow the temporal sequence of LC3 dynamics, we performed fast-iterative live microscopy under physiological and *in vitro* conditions. By performing intravital microscopy on the liver of *P. berghei*-infected *gfp-lc3* reporter mice as well as *in vitro* time-lapse microscopy of *P. berghei*-infected GFP-LC3 HeLa reporter cell line, we visualized highly dynamic LC3-positive tubular structures (Fig. 1a–d and Movies S1–S4). These structures correspond to the TVN, an extensive and highly dynamic membranous system formed by the parasite that expands the PVM into the hepatocyte cytoplasm^11^. Over the imaging period this fine-branched network evolved into multiple robust tubules, in which LC3 was steadily concentrated (Fig. 1b and Movie S2).

*In vitro* wide field microscopy performed on *P. berghei*-infected GFP-LC3 allowed monitoring the entire liver phase development of *Plasmodium*. During differentiation of sporozoites and proliferation of young schizonts, the membrane-bound LC3 is not only accumulated in the TVN, but also progressively lost from the PVM (Fig. 1d and Movie S4). *In vivo* as well as *in vitro*, the resulting mature schizonts that enter cytokinesis have cleared membrane-bound LC3 from the PVM surface. At that time, LC3 is detected in the host cytoplasm either in association with vesicles or non-lipidated (Fig. 1c,d lower panel, Movies S3 and S4). Consistently, quantification of intravital movies at different time points of the liver stage development demonstrated that >88% of the mature schizonts (40 and 44 hpi) are covered with less than 20% PVM-associated LC3 (Fig. 1e, f). While LC3 decorates sporozoites as well as young schizonts (4–24 hpi) almost entirely, it is gradually removed in proliferating schizonts (24–48 hp). Since GFP-LC3 diminishes visibly during the major growth phase of the parasite (24–48 hpi), LC3 coverage of the PVM was normalized against the parasite area defined by its cytoplasm (Fig. 1f). The disappearance of the autophagic complex from the PVM has been previously shown to positively correlate with parasite survival, while growth-restricted parasites can become a target of the host’s autodegradative pathway^7^. Indeed, our *in vivo* data confirm that more than 80% of the mature schizonts (40 hpi) become eliminated when >50% of the PVM-surface remains LC3-positive (Fig. 1g, h). The death of mature schizonts, observed on intravital movies, was defined based on morphological features and subsequent disintegration of the parasite. Three different types of parasite death have been previously characterized and are known as (i) apoptosis-like, leading to blebbing and fragmentation of the schizonts, (ii) necrosis-like, when loss of membrane integrity is observed by the influx of parasite cytoplasm into the host cell cytoplasm (Fig. 1g) and (iii) autophagy-like programmed cell death, by detecting vacuolization of parasite cytoplasm^6^. Based on this scoring, Fig. 1g represents a necrotic parasite.

Since LC3-positive parasites appear to be eliminated whereas LC3-free parasites survive and develop to infectious merozoites, we hypothesize that removal of host proteins from the PVM could represent an evasion mechanism of the parasite from the host’s selective autophagy.

### TVN expansion and plasticity contributes to the spatial control of LC3

**Figure 2:**
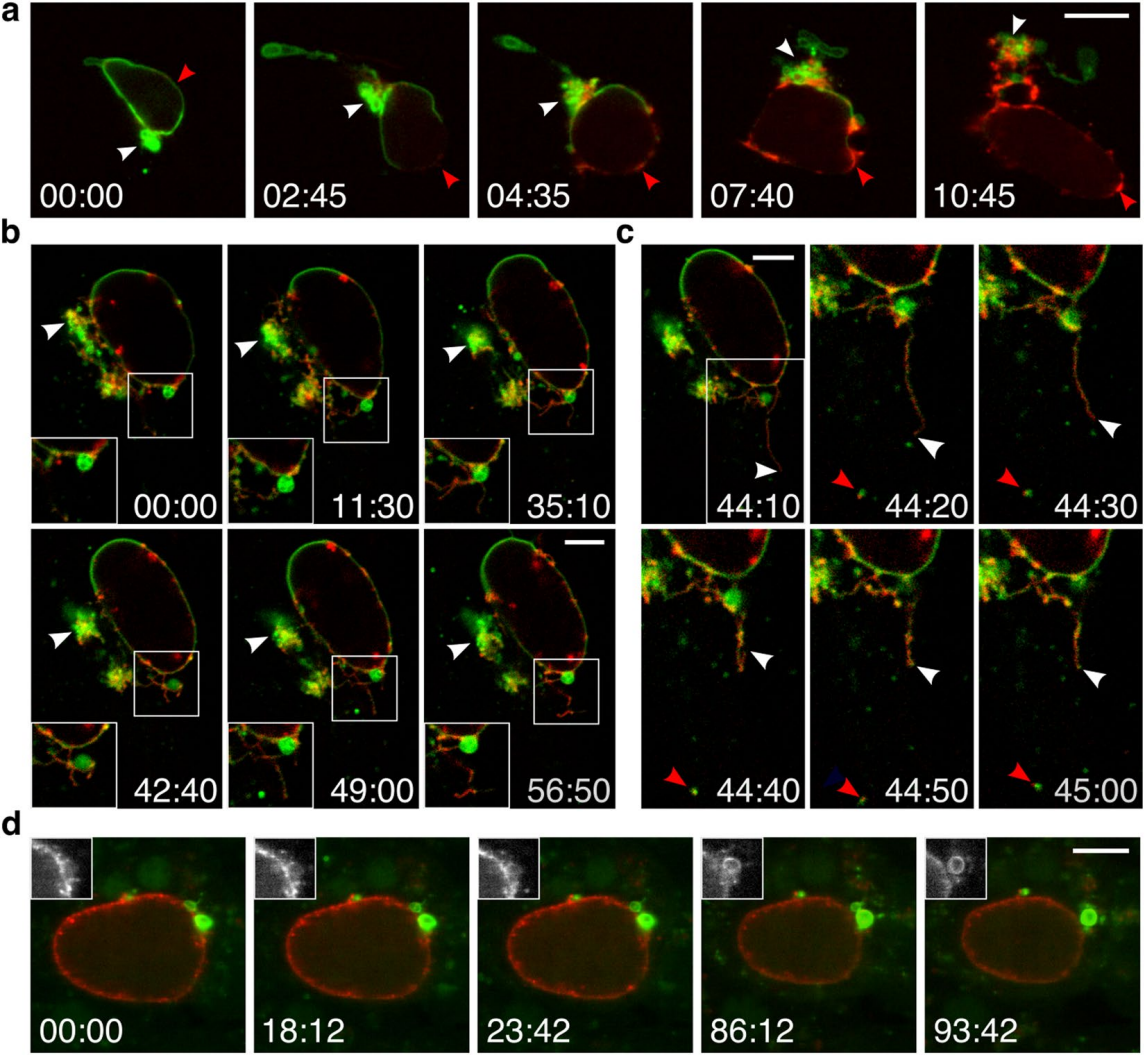
TVN expansion and dynamics promote sequestration of LC3. **(a)** Long-term imaging of GFP-LC3 HeLa cells infected with schizonts expressing EXP1-mCherry under the late liver stage promoter LISP2 (red, 33 hpi). Translocation of PVM-bound LC3 (red arrowhead) coincides with the TVN expansion (white arrowhead). Acquisition interval 5 min. **(b, c)** Fast-iterative movie of an infected GFP-LC3 cell displaying the TVN-dynamics. **(b)** A young EXP1-mCherry schizont (red, 30 hpi) shows rather static cisternae-like clusters, but motile tubules and vesicles. Arrowhead emphasizes LC3 accumulation in the TVN-cluster. **(c)** Focus on basal TVN region of the EXP1-mCherry schizonts in **(b)** shows a fast moving tubular structure (white arrowhead) and a PVM-derived LC3-vesicle (red arrowhead). Acquisition interval 10 s. **(d)** Liver intravital microscopy of *gfp-lc3* transgenic mice infected with a mature EXP1-mCherry schizont (red, 41 hpi). Residual LC3 (green) locates to the EXP1-positive TVN, highlighted in the inset. Acquisition interval 5 s. Time stamps, h:min **(a)**, min:s **(b–d)**. Scale bars, 10 µm **(a, d)**, 5 µm **(b, c)**.

The disappearance of the autophagic markers from the PVM becomes particularly pronounced during the proliferative phase of the liver-stage parasites. The massive growth of the liver schizonts is accompanied by surface enlargement of the PVM formed by the TVN. To monitor the expansion of the PVM into the TVN, long-term imaging was performed on GFP-LC3 cells infected with *P. berghei* parasites expressing mCherry fused to the PVM-resident protein Exported Protein 1 (EXP1). Endogenous EXP1 shares common TVN structures with the early PVM marker Upregulated In Sporozoites 4 (UIS4) (Fig. S1a) and parasite-associated LC3 (Fig. S1b) already in young schizonts (24 hpi). Since the exogenously tagged EXP1-mCherry is driven by the late liver stage-specific promoter LISP2^28, 29^, expression starts to increase from 30 hpi onwards. During schizont maturation EXP1-mCherry localization shifted into the existing, LC3-positive TVN. Concomitant with expansion of the TVN, LC3 became steadily removed from the PVM (Fig. 2a and Movie S5).

Since the various morphological features of the TVN have been associated with distinct dynamics^11^, we recorded short-term movies with high frequency intervals. Fast-iterative imaging of *P. berghei*-EXP1-mCherry showed a substantial fraction of LC3 accumulating in the large cisternae-like TVN clusters (Fig. 2b and Movie S6). While the large cisternae-like TVN clusters appear more passive, the tubular protrusions behave highly motile, demonstrating similar dynamics as the two previously described PVM-marker, UIS4 and intra-erythrocytic *P. berghei*-induced structure 1 (IBIS1)^11^. Over the imaging period, the highly dynamic tubular protrusions of the young schizont (30 hpi) generate a densely branched mesh (Fig. 2b, c and Movie S6). The same degree of TVN-plasticity can be observed in young schizonts expressing the fluorescently-tagged early PVM-marker UIS4 (Fig. S2a and Movie S7). Aside of its dynamic behaviour at the TVN, LC3 concentrated in several patches at the PVM, which progressively moved into the TVN (Figs 2b, c and S2b, Movies S6 and S8). Yet, TVN protrusions from mature schizonts were considerably shorter and less motile than those in earlier stages (Fig. 2d and Movie S9). Additionally, residual LC3 in mature schizonts remained restricted to large individual clusters corresponding to the EXP1-positive TVN (Fig. 2d and Movie S9). Progressive accumulation of LC3 in the TVN strongly correlates with TVN-dynamics and suggests a directional membrane shedding, potentially reducing the overall load of LC3 at the PVM.

### The TVN resembles a cellular trap for host-autophagic proteins

**Figure 3:**
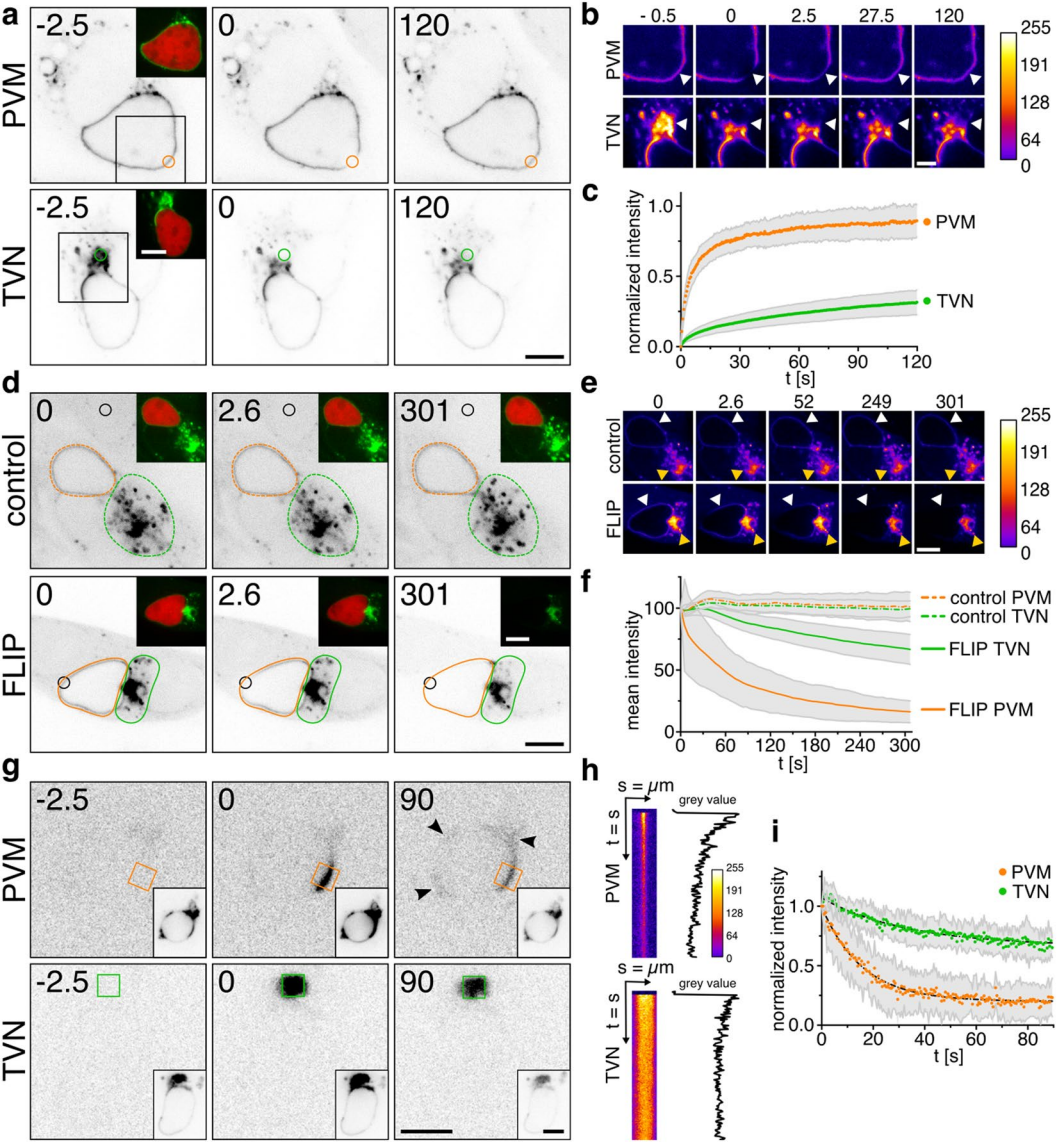
Compartment-specific LC3 mobility reveals trapping of LC3 in the TVN. **(a–c)** Fluorescence Recovery After Photobleaching (FRAP) of GFP-LC3 HeLa cells infected with *Pb*mCherry 33-36 hpi. Fluorescence recovery reveals a distinctive LC3 motility at the PVM (orange ROI) versus TVN (green ROI). **(b)** Pseudo-coloured heat maps visualizing the dynamics of LC3-recovery at the bleached area (arrowhead). **(c)** FRAP-quantification at the PVM (orange) and the TVN (green) (n = 40; mean ± s.d.). **(d–f)** Fluorescence Loss In Photobleaching (FLIP) shows loss of LC3 at the PVM and retention at the TVN. **(d)** Repetitive bleaching (black ROI) performed on an adjacent, uninfected GFP-LC3 cell for imaging-induced fluorescence decay (control), or on PVM-localized LC3 (FLIP). Mean fluorescence intensity of GFP-LC3 measured around PVM (orange ROI) and TVN (green ROI). **(e)** Pseudo-coloured heat maps of GFP-LC3 intensity at the PVM (white arrowhead) or TVN (yellow arrowhead). **(f)** FLIP-quantification at the PVM (orange) and TVN (green) (n = 40; mean ± s.d.). **(g)** Photoconversion of Dendra2-LC3 at the PVM (orange ROI) or TVN (green ROI) showing pre-activation, post-activation and final image of young schizonts (24 hpi, *Pb*wt). Insets display the green channel (non-converted Dendra2-LC3). Arrowheads point out the re-distribution of converted Dentra2-LC3 at the PVM. **(h)** Representative, pseudo-coloured kymograph of activated LC3 in **(g)** measured at ROI. **(i)** Quantification of LC3 at the PVM (orange) and TVN (green) based on kymographs (n = 10; mean ± s.d.). Time stamps in s. Scale bars, 10 µm **(a, d, g)** and 5 µm **(b, e)**.

Since compartmentalization of the PVM could be associated with different membrane properties and thus influence the mobility of LC3, we compared the diffusion pattern of GFP-LC3 at the PVM and TVN of young schizonts. Fluorescence recovery after photo-bleaching (FRAP) revealed a compartment-specific mobility of LC3, with significantly slower recovery rates at the TVN. While LC3 moved freely in the PVM, its mobility was impaired in the tangled architecture of the TVN (Fig. 3a–c and Movie S10). Moreover, repetitive bleaching of LC3 at the PVM, by applying fluorescence loss in photo-bleaching (FLIP), led to a loss of 87% of the mean fluorescence intensity of PVM-associated LC3. Although the TVN is connected to the PVM, the GFP-LC3 fluorescence was mostly retained in the TVN and potentially trapped in the convoluted network (Fig. 3d–f and Movie S11).

In order to track and quantify the kinetics of the parasite-associated LC3, we fused the autophagic marker to the photo-convertible fluorescent protein Dendra2. Activation with a short 405 nm laser pulse irreversibly converts Dendra2 from a green to a red-emitting state^30^. After photo-switching of a defined pool of Dendra2-LC3 either at the PVM or TVN, its redistribution was quantified by space-time segmentation over a period of 5 min (Fig. 3g–i). Consistent with our photo-depletion experiments, activation of Dendra2-LC3 at the PVM demonstrated a rapid diffusion of LC3 with only 20% left at the original activation site. This highly dynamic lateral exchange of LC3 within the PVM could potentially facilitate the exit into the TVN, where 66% of the converted Dendra2-LC3 remained immobile (Fig. 3h, i). The distinct LC3 dynamics suggest that the TVN functions as a subcellular trap, where host autophagic proteins are disposed of in a safe distance from the parasite’s replicative centre.

**Figure 4:**
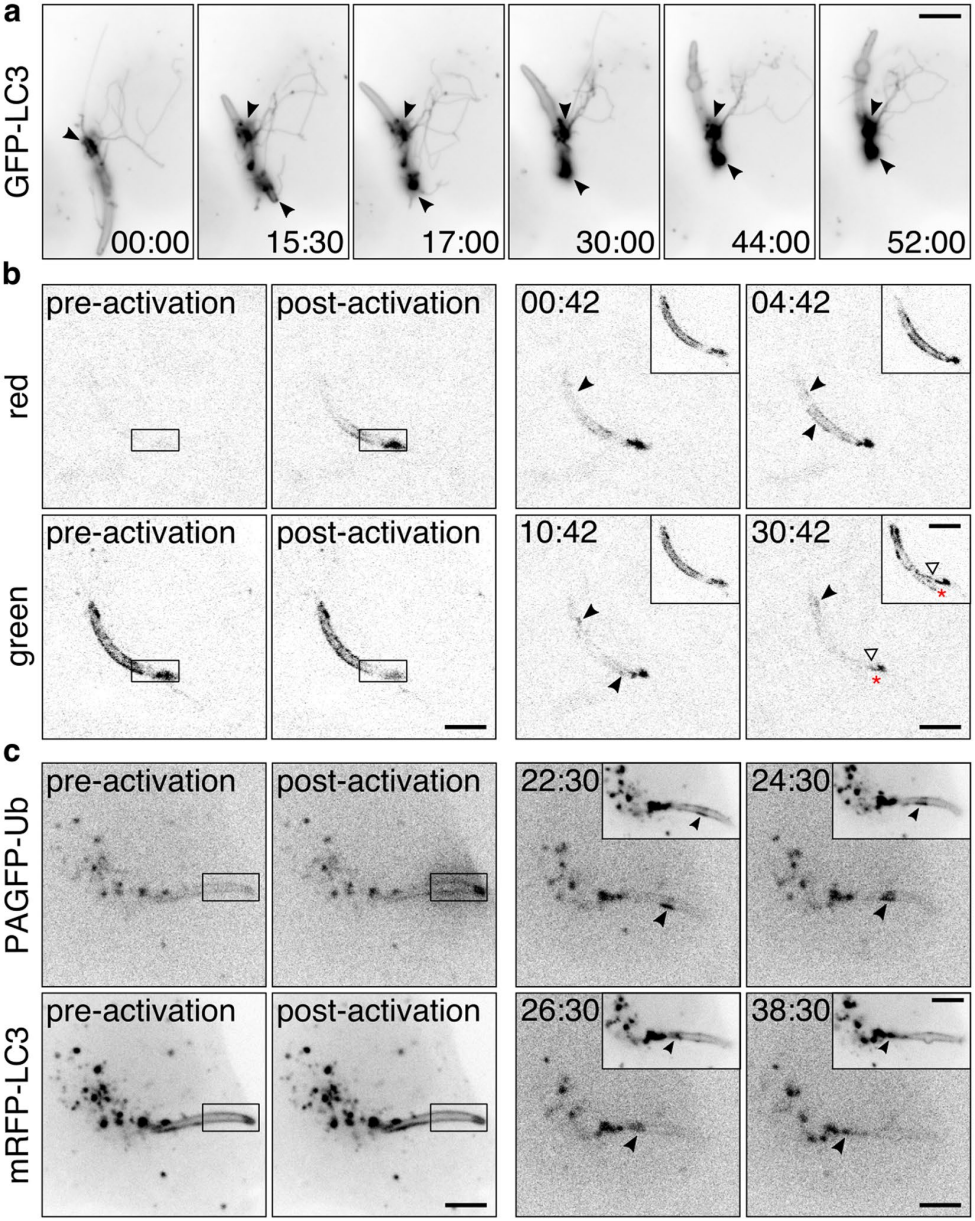
Control of the autophagic components coincides with TVN development. **(a)** Fast-iterative wide field microscopy of *Pb*mCherry sporozoites (1 hpi) outlined by parasite-associated LC3. Arrowheads indicate LC3-accumulation in the TVN. Acquisition interval 30 s. **(b**) Photoconversion of Dendra2-LC3 at the tip of a *Pb*wt sporozoite (3 hpi) comprising a nascent TVN. Activated LC3 (black ROI) is tracked at the PVM and accumulates in membrane patches (black arrowhead). White arrowhead points out the accumulation of non-activated LC3 in the TVN. Red asterisk indicates the tip of the sporozoite. Inset displays non-activated LC3 in the green channel. **(c)** Photoactivation of PAGFP-Ub at the PVM of sporozoites (2 hpi, black ROI) in HeLa cells co-transfected with pmRFP-LC3. Activated ubiquitin (Ub) and LC3 share patches and relocate together from the anterior tip into the posterior TVN-accumulation (black arrowhead). Insets display mRFP-LC3. Time stamps, min:s. Scale bars, 5 µm.

The nascent PVM is formed over the course of invasion by invagination of the host plasma membrane^31^ and becomes rapidly decorated with lipidated LC3^7–9^. Spatial control of LC3 could facilitate the sporozoite’s escape from the host surveillance system. Indeed, during the first hour of invasion by the sporozoite, the LC3-positive TVN evolved into a fine-branched network (Fig. 4a and Movie S12), showing similar dynamics as the previously described PVM-resident protein UIS4^11^. Moreover, we observed the development of multiple LC3-accumulating TVN clusters. Based on the high temporal resolution of fast-iterative wide field microscopy, emergence of the sporozoite’s TVN could aid in the redistribution of LC3 and restrict its quantity at the PVM.

To test if sporozoites redirect and deposit the PVM-targeted LC3 into the connected TVN, we monitored photo-converted Dendra2-LC3 over an imaging period of 1 h. Sporozoites showed a fast lateral diffusion of photoactivated LC3, assembling into multiple membrane patches at the PVM (Fig. 4b and Movie S13). Additionally, the existing TVN cluster developed a nascent tubular protrusion pushing the LC3 accumulation away from the sporozoite. Analogous observations were made in infected HeLa cells co-transfected with mRFP-LC3 and photoactivatable GFP-ubiquitin (PAGFP-Ub) (Fig. 4c and Movie S14). Ubiquitin (Ub) is known to interact through the adaptor protein SQSTM1/p62 with LC3 during selective autophagy^10, 32^ and it has been previously shown that it becomes cleared from the PVM surface with similar temporal dynamics as LC3^7^. A defined region of interest (ROI) located distal from the sporozoite’s TVN was photoinduced to convert PAGFP-Ub from its dark to a green fluorescence state. Both mRFP-LC3 and photoactivated Ub assembled into identical membrane patches that subsequently exited into the TVN located at the posterior end of the sporozoite (Fig. 4c and Movie S14). Since various PVM-associated host proteins demonstrate same dynamics, their spatiotemporal control seems to reflect an intrinsic mechanism of the PVM.

### Vesicle shedding of host-autophagic proteins into the host cytoplasm

**Figure 5:**
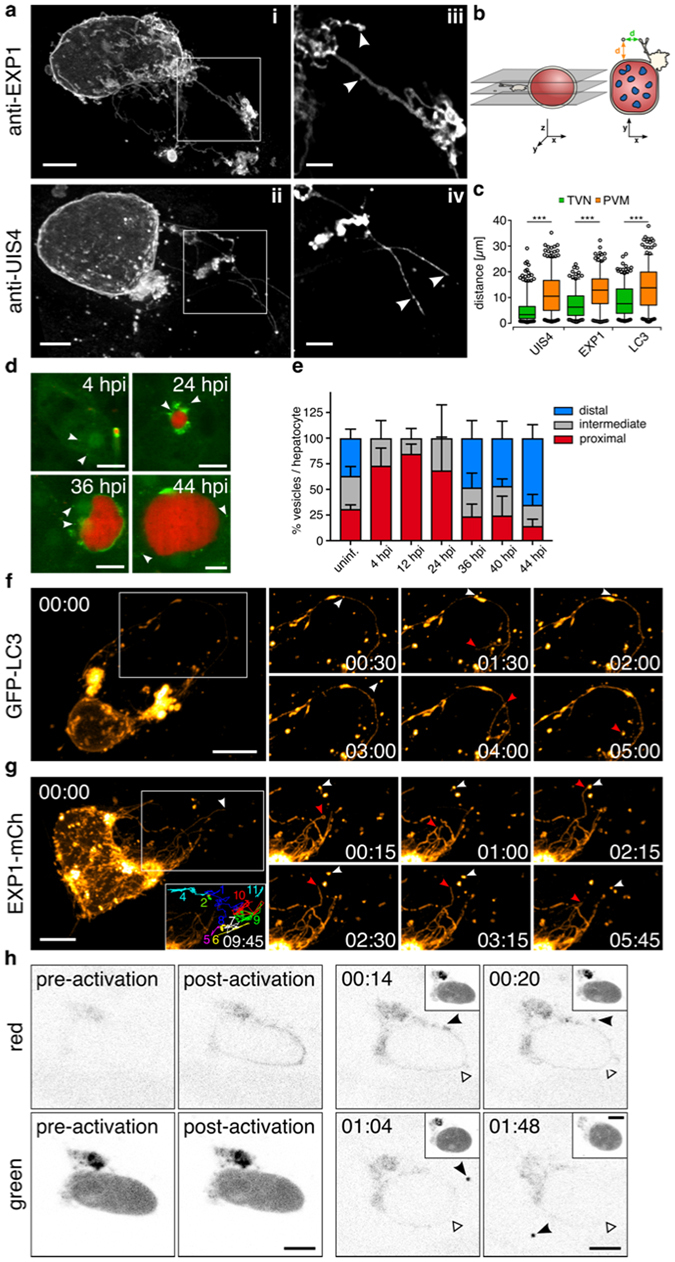
TVN-architecture and vesicle shedding in liver schizonts. **(a)** 3D reconstructions of the PVM/TVN architecture. Young liver schizonts (36 hpi) were labelled for the indicated PVM proteins by immunofluorescence and images acquired by confocal laser scanning microscopy (3D-CLSM) with 0.22 µm z-increments. The TVN consists of branched tubular structures, several node-like clusters and vesicles. **iii, iv**, Magnifications of a single z-plane. Potential budding sites or “growth cones” (arrowheads) are visible along protrusions and TVN-clusters. **(b**,** c)** The origin of the parasite-derived vesicles was determined based on the scattering pattern in the infected host cell. **(b)** Schematic of the analysis performed on *Pb*mCherry schizonts (36 hpi) either stained for UIS4 or EXP1. LC3 vesicles were determined on GFP-LC3 HeLa cells infected with *Pb*mCherry. **(c)** Quantification of the minimal distance of PVM-/LC3-vesicles from the TVN (green) and PVM (orange). n > 200 vesicles. ***P < 0.0001, two-way ANOVA. **(d, e)** *In vivo* quantification of the mean LC3-vesicle distribution. **(d)** Representative images from intravitals performed on the liver of *gfp-lc3* mice infected with *Pb*mCherry at different stages of the infection. **(e)** The percentage of vesicles scored within a defined area relative to the parasite surface, represented in a stacked column graph. Host cytoplasm of infected hepatocytes was divided into 3 consecutive sections, referred to as proximal, intermediate and distal. **(f, g)** Fast iterative 3D-movies displaying TVN dynamics. **(f)** GFP-LC3 positive schizont (24 hpi) recorded with 0.25 µm z-increments and 30 s intervals. White arrowhead highlights formation and budding of a LC3-vesicle. Red arrowhead tracks a LC3-positive protrusion. **(g)** *P. berghei-*EXP1-mCherry schizonts 33 hpi recorded with 0.4 µm z-increments and 15 s intervals. Inset shows maximum projection of tracked vesicles. Arrowheads highlight the dynamics of an EXP1-protrusion (red) and EXP1-vesicle (white). **(h)** Photoconversion of Dendra2-LC3 at the PVM of *Pb*GFPcon schizonts (33 hpi). Converted LC3 accumulates in a vesicular structure (black arrowhead), which is shed from the PVM. At the same time converted LC3 is lost from the original activation site (white arrowhead). Insets display the green channel (parasite cytoplasm and non-converted LC3). Time stamps, min:s **(f, g)** and h:min **(h)**. Scale bars, 10 µm **(d, f, g)**, 5 µm **(h**, i, ii) and 2 µm (iii, iv).

Since our data suggest an involvement of the TVN in spatially restricting PVM-associated host factors, we aimed to gain further insight into the morphological features of the membrane network. To resolve its micro-architecture in three-dimensions (3D) we stained schizonts against endogenous PVM-resident proteins, such as EXP1 and/or UIS4. High-resolution 3D confocal laser scanning microscopy (3D-CLSM) revealed a bipartite organization of the TVN in cisternae-like clusters and tubular protrusions. Numerous PVM-derived vesicles in the host cytoplasm surround these large clusters. While cisternae-like clusters resemble potential blebbing membranes, the protrusions harbour additional putative exit sites for budding vesicles, or branching points for nascent tubules (Figs 5a and S1a). In GFP-LC3 cells, the multicolour 3D reconstructions of the PVM demonstrated numerous cytosolic LC3-positive vesicles containing parasite-derived PVM proteins, potentially resulting from vesicles shed off the vacuolar membrane (Fig. S1b).

The compartmentalization of the PVM and TVN might also define the origin of these vesicles. Distance measurements from both PVM-derived and LC3-positive vesicles to the vacuolar surface of young schizonts (36 hpi) were performed on z-slices of 3D images (Figs 5b and S3a). The median distance between vesicles and the TVN (<10 µm) was significantly shorter than to the PVM. Thus the TVN could serve as the main shedding compartment (Fig. 5b, c). Interestingly, when the average displacement of LC3-vesicles in relation to the parasite’s surface was quantified *in vivo*, we observed a characteristic pattern and distinct vesicle gradient at different times of infection. While in sporozoites and young schizonts (4h-24h) LC3-vesicles are found exclusively in the parasite’s proximity, the distribution significantly shifted toward the host cell periphery in late stages. Although the decrease in the host’s cytoplasmic space in mature schizonts represents a limiting factor in this assay, we observed the same tendency in spacious areas of the infected hepatocyte. In uninfected hepatocytes, however, LC3-positive autophagosomes scatter randomly in the cytoplasm in relation to the host nucleus (Fig. 5d, e). In *in vitro* assays, the total number of LC3 vesicles in infected HeLa cells was significantly higher compared to the PVM-derived vesicles (Fig. S3). This could be a consequence of active host autophagy machinery.

In order to discriminate between host autophagosomes and potential membrane shedding, we performed 3D-movies of LC3-targeted young schizonts. With this approach we were able to capture the formation and subsequent fission of LC3 vesicles from the TVN (Fig. 5f and Movie S15). By tracking PVM-derived vesicles during 3D time-lapse microscopy of schizonts expressing EXP1-mCherry, we observed highly motile vesicles. Although individual vesicles seemed to transiently interact with TVN extensions and showed potential homo-fusion between vesicles, no back-fusion with the TVN was recorded (Fig. 5g and Movie S16). Thus the observed unidirectional vesicle shedding from the TVN into the host cytoplasm ensures spatial separation.

The manifestation of PVM-derived, LC3-positive vesicles (Figs 2b and S1, Movie S6), in addition to the observed LC3 budding (Fig. 5f and Movie S15), suggests that trapping of the host determinants in the TVN might not represent their final destination. Especially during schizont maturation the number of LC3-vesicles in the host cytoplasm significantly increases^7^. To follow a particular LC3 shedding event in a developing schizont, we photoconverted PVM-associated Dendra2-LC3 at a ROI opposite of the TVN. The photoconverted LC3 at the PVM gradually disappeared and accumulated in the TVN (Fig. 5h and Movie S17). In addition, a fraction of LC3 accumulated at a TVN junction, which further condensed to a vesicle-like structure and was finally shed into the host cytoplasm. Therefore, elimination of host autophagy proteins from the PVM surface appears to involve not only gathering in the TVN, but ultimately shedding of host factors back to the host cytoplasm.

## Discussion

**Figure 6:**
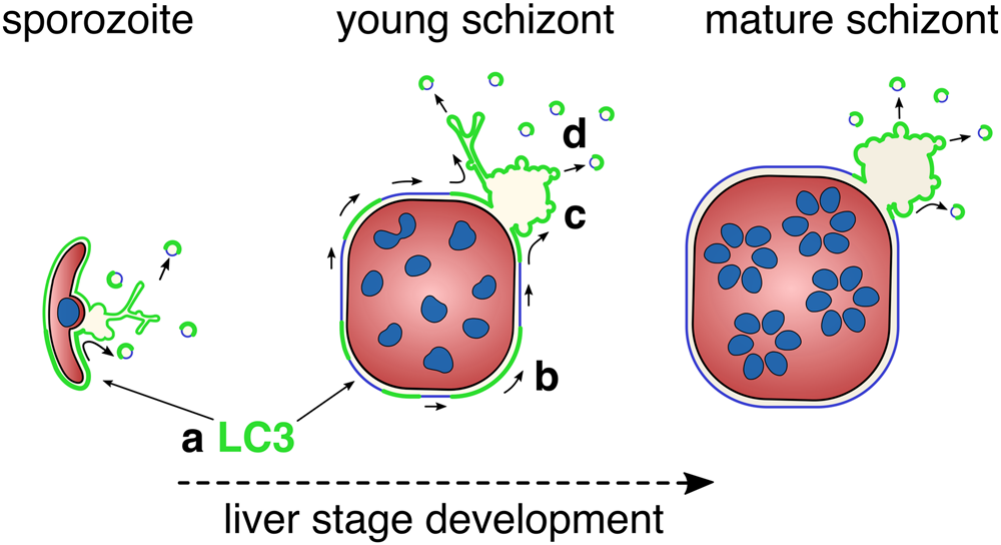
Model of the putative shedding mechanism during the hepatic stage of *P. berghei*. Progressive removal of PVM-associated LC3 during the *P. berghei* liver stage development. **(a)** LC3 incorporation into the PVM, **(b)** accumulation into PVM-patches, **(c)** transfer and entrapment in the TVN, and **(d)** vesicle shedding into the host cytoplasm.

Selective autophagy is an important first-line cell response, facilitating cross-recognition and elimination of different *Plasmodium* species in hepatocytes^7, 8, 18^. Nevertheless, host autophagic and lysosomal markers are lost from the PVM of mature liver-stage *P. berghei* schizonts. Loss of autophagy marker proteins from the PVM positively correlates with successful completion of the parasite liver stage and implies a putative evasion strategy^7^. Our present quantitative live imaging study provides evidence that clearance of these marker proteins from the PVM is mediated by an active membrane shedding process, rather than an *in situ* proteolytic cleavage of the lipidated LC3 through the Atg4B de-conjugation pathway^33^. We further demonstrate that the TVN functions as a subcellular trap, where host autophagic proteins are temporarily sequestered before becoming shed into the host cytoplasm by vesicle fission (Fig. 6). Thus, PV membrane shedding and vesicle formation represents a potential self-defence strategy of the malaria parasite, to escape a cytosolic innate immune response in non-immune cells.

In extracellular parasites that are exposed to their host’s humoral response, the removal of these surface-bound host mediators is a common feature. *Trypanosoma brucei* for example, besides altering its antigenic surface coat, clears surface-bound immunoglobulins through endocytosis at the flagellar pocket^34, 35^. Likewise, antigen-antibody complexes (surface receptor caps) on the *Entamoeba histolytica* surface become shed into the extracellular milieu, providing resistance against the complement-mediated system of the host. These surface-targeting antibodies are first deposited in the uroid region (posterior bulb) and spontaneously released as macromolecular aggregates (capping)^36, 37^. We show that the intracellular pre-erythrocytic stages of *P. berghei* have evolved an analogous, spatiotemporal control of PVM-targeted autophagy proteins, to subvert an autophagy-mediated turnover of the parasite. The PVM-associated host factors become (i) assembled into membrane patches at the PVM, (ii) deposited and trapped in the TVN, and finally (iii) shed as vesicles into the host cytoplasm (Fig. 6). This mechanism could exemplify an alternative strategy, distinctive from other intracellular pathogens that escape from the autophagic-mediated surveillance by either blocking or hijacking a certain step in the pathway or by evading autophagic recognition. In the related apicomplexan *T. gondii*, strain-specific effector proteins circumvent IFNγ–dependent recruitment of ubiquitin and autophagic adaptor complex as well as the ubiquitin-like conjugation system to the PVM^18, 19, 38^. Avirulent strains of *T. gondii* failing to control this autophagy machinery become eliminated^38^. Therefore, these *T. gondii*-specific effectors represent critical virulence factors, affecting natural host resistance and a possible strain-specific host predilection.

In case of *P. berghei*, the nascent PVM of sporozoites is recognized by an autophagy-related mechanism within the first hour after invasion. At the same time, the PVM is modified to a customized and adaptable exo-parasitic organelle with a highly compartmentalized network, the TVN^11^. Compositional modifications of the PVM arise from a general export of parasite proteins and could represent a potential driving force for the directional movement of autophagic proteins. Moreover, the TVN steadily expands and remains dynamic during the entire growth phase of the parasite. Based on our photoconversion experiments using Dendra-LC3 and PAGFP-Ub, development of the TVN appeared to mobilize the host autophagy complex at the PVM. The segregation of the PVM-located autophagic complex into multiple, microscopically visible patches seem to be a result of fast lateral movement. The lipid composition of the PVM could contribute to the organisation of LC3-patches in the PVM. A potential mediator may be cholesterol, which is well-recognized for coordinating the assembly of membrane microdomains like lipid rafts^39^. Indeed, host-derived cholesterol is found enriched in the PVM of *Plasmodium* ^40, 41^. Since these LC3-positive patches appear to move unrestricted in the PVM, and to remain highly motile, they can eventually exit into the TVN. This spatial displacement of the host determinants could protect the integrity of the PVM to ensure parasite survival.

The digestive capacity of autophagy depends on the fusion with lysosomes. Since interference with the endolysosomal pathway affects parasite proliferation^41–43^, *Plasmodium* liver stages appear to not actively impede autophagosomal maturation. Both the functional autophagy machinery as well as the endolysosomal pathway represent a critical route to scavenge essential nutrients such as lipids and amino acids from the host cell^7, 9, 41–43^. Lysosome recruitment to the PVM appears orchestrated by the induction of the LC3-associated phagocytosis (LAP)-like pathway^8^ as well as by autophagy-independent mechanism^41^. In *P. berghei*, endocytic cargo was visualized in the PV lumen, demonstrating the fusogenic potential of the PVM^42^. Nevertheless, in cells harbouring viable parasites, acidification of the PV lumen is possibly prevented by the presence of non-selective channels. Interestingly, cholesterol has been proposed to modulate permissiveness of these channels^40^. Additionally, lysosomes in infected host cells show similar spatiotemporal changes at the PVM such as LC3^7^. By trapping PVM-associated host proteins in the convoluted mesh of the TVN, the parasite could protect its vulnerable replicative centre. Especially, the large immobile cisternae-like clusters appear to preferentially concentrate autophagic markers. These spatial constrictions might contribute to the maintenance of a stable vacuole environment and simultaneously ensure efficient nourishment of the parasite.

A particular role of the exomembranous system in immune evasion had only been characterized for the pathogenic erythrocytic parasite stages. Here, *Plasmodium* functionalizes this membranous structure into a homemade secretory organelle, to export cytoadherent proteins through the red blood cell cytoplasm onto its surface. The resulting tissue-specific sequestration facilitates the parasite’s escape from spleen-mediated clearance^44–46^. Interestingly, erythrocytic stages possess more morphologically distinct exomembrane structures, which can be differentiated into PVM, TVN, Maurer’s clefts (individual membrane-bound sacks) and vesicles. In contrast to blood stage parasites, liver stages generate a much more pronounced and extensive TVN^44^. Our 3D-image analysis revealed a very complex micro-architecture of the TVN and a less uniform morphology between different infected hepatocytes. Although extensively studied in the erythrocytic stage, the molecular machinery involved in the formation of the exomembrane structure remains elusive. It is likely that the same key players are involved in the shedding process observed in the present work. Based on the resolved TVN micro-architecture and observed vesicle budding, the TVN appears to serve not only as a primary disposal area but to represent the major shedding site. Ultimately, vesicle shedding guarantees the definite spatial separation of the autophagic proteins from the PVM. PVM proteins localized in vesicles such as UIS4, IBIS^11^ and LISP2^47^ have been described previously, but not in the context of host protein shedding. Since the number of PVM-derived vesicles in the host cytoplasm varies strongly^11^, shed material could be subject for a fast degradation. In fact, liver stage parasites are shown to restrict export of parasite-derived material in order to prevent MHC-I presentation, and priming of effector CD8+ T cells^48^. Likewise, shed vesicles could decoy autophagic machinery and even stimulate the autophagic turnover of the host cytoplasm. The recycled macromolecules could further nourish the parasite. Indeed, supporting previous observations in *T. gondii*, host canonical autophagy enhances growth of *P. berghei* liver stages^7, 9, 49^. Thus, *Plasmodium* liver stages seem to persist within, and take advantage of an autophagically active host environment and simultaneously escape the hostile, intrinsic host defence.

In addition to the importance of autophagy in nutrient homeostasis, its selective degradation pathway plays a critical role in the host’s intracellular defence. This pleomorphic function of autophagy is nicely reflected by its ambivalent role in *P. berghei* liver stage development. Further studies are required to better discriminate the molecular signature involved in the specific elimination of *Plasmodium* liver stages through selective autophagy and the nourishing capacity of host canonical autophagy.

## Material and Methods

### Animal Work Statement

Experiments performed on laboratory animals were in accordance with European animal regulations and approved by the ethical committee of the University of Bern (Permit # BE109/13 and BE81/11).

*Plasmodium berghei* infections were conducted in 6 to 10 weeks old Balb/c mice, either bred in the central animal facility of the University of Bern or supplied by Harlan Laboratories. Intravital microscopy was performed in *gfp-lc3* C57BL/6N transgenic mice aged from 6 to 8 weeks and were kindly supplied by Noboru Mizushima^50^.

### Plasmid Construction

For the transient expression of the photoconvertable fluorescent LC3 fusion protein, the N-terminal *egfp* was replaced by *dendra2* in the pEGFP-LC3 (kindly provided by Jonathan C. Howard) using the NheI and XhoI restriction sites. The *dendra2* was PCR amplified from the pDendra2 plasmid using the oligonucleotides 5′-TTGCTAGCATGAACACCCCGGGAATTAACC-3′ and 5′-TTCTCGAGTAACACCTGGCTGGGCAGG-3′. The final plasmid was named pDendra2-LC3.

Further plasmids utilized in this study were PAGFP-Ub (addgene plasmid #11933) and pmRFP-LC3 (addgene plasmid #21075).

Stable cell lines were generated using the lentivirus system. The VSV-G envelope expressing plasmid pMD2.G (addgene plasmid #12259), the 2^nd^ generation packaging plasmid psPAX2 (addgene plasmid #12260) and lentiviral expression plasmid pRRLSIN.cPPT.PGK-GFP.WPRE (addgene plasmid #12252) were kind gifts from Didier Trono.

For generating pRRL.EGFP-LC3 and pRRL.Dendra2-LC3 lentiviral vector plasmids the full expression cassette was PCR amplified using the oligonucleotides 5′-TACGGACCGATGGTGAGCAAGGGCGAG-3′ (*egfp*) or 5′-ATCGGACCGGCTAGCATGAACACC-3′ (*dendra2*) in combination with the oligonucleotide 5′-ATCGGTCCGTTACACAGCCATTGC-3′ binding to the C-terminus of *LC3*. The cassettes were ligated in to the pRRLSIN.cPPT.PGK-GFP.WPRE through the RsrII restriction site replacing the original e*gfp* open reading frame (ORF).

### Cell Culture and Infection of Mammalian Cells

For tissue culture of human epithelial carcinoma HeLa cells (a kind gift from Robert Ménard) and human embryonic kidney cell line HEK293T (ATCC) were cultivated in Minimum Essential Medium with Earle’s salts (MEM) or Dulbecco’s Modified Eagle Medium (DMEM), each supplemented with 10% FBS, 2 mM L-Glutamine and 100 IU/ml penicillin and 100 µg/ml streptomycin (all from PAA Laboratories) in a humidified 37 °C and 5% CO_2_ incubator.

Infections were performed with either *P. berghei* ANKA wild type (*Pb*wt) strain or the transgenic strains *Pb*GFPcon (RMgm-5, URL: www.pberghei.eu), *P. berghei* mCherry(hsp70) (RMgm-928, URL: www.pberghei.eu), *P. berghei-*EXP1-mCherry^51^ and *P. berghei* UIS4-mCherry^11^. For *in vitro* infections of HeLa cells, sporozoites were isolated from salivary glands of female *Anopheles stephensi* mosquitos 16 – 27 days after blood feeds. A confluent layer of HeLa cells was inoculated with approximately 5 × 10^4^ sporozoites and reseeded 2 hpi onto a respective cell culture dish containing 0.25 µg/ml Amphotericin B (PAA laboratories), at the same time terminating any further invasion.

### Transient Transfection of Mammalian Cells

Transient transfections of HeLa cells were performed using the Nucleofector Electroporation Technology (Lonza). Cells were seeded the day before to ensure exponential growth and harvested by Accutase treatment. For a single transfection 0.5–1 × 10^6^ cells were resuspended in Nucleofector V solution (Lonza) and transfected with 1–2 µg plasmid DNA using the program T-28.

### Lentiviral Production and Transduction of Mammalian Cells

Lentiviral particles were produced by transient transfection into HEK 293 T cells as described by the laboratory of Didier Trono (http://tronolab.epfl.ch/lentivectors). A total of 2.5 × 10^6^ cells were seeded in T75 flask the day before and the culture medium was changed 2 h prior to transfection containing no antibiotics. Transfections were performed with a total 30 μg of plasmid proportion of 1:2:3 (envelope: packaging: vector plasmid) using the calcium phosphate co-precipitation method. The medium was replaced 12 h post transfection and the conditioned medium harvested after 24 h, 48 h and 72 h. The viral supernatant was cleared from detached cells and debris by 500 × g centrifugation and filtering through 0.22-μm-pore-size cellulose acetate filters.

For the production of stable HeLa transgenic cell lines, exponentially growing target cells were transduced with virus supernatant at three consecutive times. Expression of the transgene was assessed by fluorescence microscopy 48 h after transduction and the single clones isolated by limiting dilution.

### Indirect Immunofluorescence Analysis and Three Dimensional (3D) Visualisation

Infected HeLa cells were fixed with 4% (w/v) paraformaldehyde in PBS and subsequently permeabilized with 0.1% Triton-X 100 each for 20 min at room temperature. After blocking non-specific sites with 10% FBS for 1 h at room temperature, cells were incubated with a polyclonal rabbit anti-UIS4 (kind gift from Photini Sinnis), polyclonal chicken anti-EXP1 (generated by Heussler lab) each diluted 1:500 and/or 1 µg/ml monoclonal mouse anti-GFP (Roche) as primary antibody. In a second step the samples were labelled with 0.7 µg/ml of a species-specific Alexa Fluor 488 IgG (H+L), Alexa Fluor 594 IgG (H+L) (both form Molecular Probes) and Cy5 IgG (H+L) (Jackson Immuno Research) fluorophore-conjugated antibodies, respectively. The nuclei were visualized using 0.2 µg/ml of the DNA dye 4′,6-Diamido-2-Phenylindole (DAPI; from Invitrogen). Specimens were embedded in Mowiol (Roth) containing 2.5% DABCO (Roth) antifade.

Higher resolution multicolour microscopy was conducted on a Leica TCS SP8 equipped with a HC PL APO CS2 63 × 1.4 NA immersion oil lens, a 405 nm Diode laser and a white light laser (WLL), and a tuneable accustom-optical beam splitter (AOBS). To avoid any cross talk between the channels, images were collected in line sequential mode with a dwell time of 1.1 µs and z-increments of 0.22 µm. Images were further processed using Huygens deconvolution software and the subsequent 3D volumes were generated using the Maximum Intensity Projection (MIP) rendering method from SVI (Netherlands).

### Vesicle Quantification on z-Stack Images

For determining the total number of vesicles per infected cell as well as distance of vesicles from the PVM and TVN, images were obtained on the Andromeda confocal spinning disc system from Till Photonics (FEI Munich) equipped with an APO 60 × 1.35 NA immersion oil Olympus objective and an additional 1.22x magnification lens in front of Andor 897 high speed EMCCD camera. For z-Stacks with increments of 0.15 µm the Alexa Fluor 488 and mCherry were consecutively excited for 100 ms with 488 nm and 561 nm laser and spectral separated by a 525/50 nm Bright Line single-band bandpass and 594 LP Edge Basic long-pass filter, respectively. Images were deconvolved using Hyugens deconvolution software (SVI). The centre of the vesicles was manually determined based on the maximum intensity and radius and subsequently marked using the FIJI point tool. The minimal distance of each vesicle to the TVN and PVM was measured on individual stacks and plotted in a box plot with 5 and 95 percentiles shown for individual values. *P*-values were determined by performing a two-way ANOVA. Same images were used to determine the total number of vesicles per infected cell. Values are plotted in a box plot and *P*-values were determined by performing a one-way ANOVA.

### Time-lapse Microscopy

For real time microscopy on invaded sporozoites or young trophozoites, 10^5^ HeLa cells were seeded onto glass bottom dishes (MatTek) and inoculated the next day with multiplicity of infection (MOI) of 3. Live cell imaging of later stages such as schizonts involved reseeding of HeLa cells on a glass bottom dish (MatTek) 4 h post-inoculation with sporozoites to avoid over-confluency of the host cells.

Wide field microscopy was conducted on an inverted Leica DMI600B equipped with a climate chamber using a HCX PL APO 63×/1.2 water immersion objective with an automated water micro-dispenser for long-term live cell imaging or a HCX PL APO 100×/1.4 oil immersion objective for the fast iterative time-lapse microscopy. Images were recorded with Leica DFC 365 FX camera (Leica Microsystems, Wetzlar, Germany).

Confocal microscopy was conducted on the previously described inverted Leica TCS SP8 under temperature and CO_2_ controlled environment using the HC PL APO CS2 63×/1.4 NA immersion oil objective. For fast data acquisition, dual fluorescence was detected simultaneously using the 8.000 Hz resonant scanner with 32–64 times frame averaging and a minimum cycle time of 10–20 s per frame. GFP and mCherry was excited with the 488 nm or 590 nm laser line and the emission spectra detected with the AOBS ranging of 500–550 nm and 600–800 nm, using the hybrid detectors, respectively. The recorded movie 6 was bleach corrected by histogram matching using FIJI software (URL: http://fiji.sc/Fiji)^52^.

High-speed 3D time-lapse microscopy was performed on the Andromeda confocal spinning disc system from Till Photonics (FEI Munich) with an environmental chamber from Ludin (Life Imaging Services, Switzerland). Images were acquired with a step size of 250 or 400 nm using the APO 40×/1.35 NA immersion oil Olympus objective and an additional 1.2x magnification lens mounted in front of the Andor 897 high-speed EMCCD camera. GFP was excited with a 488 nm laser and collected through a 525/50 (BrightLineHC) filter. mCherry was excited at 561 nm and filtered through a 594 LP Edge Basic long-pass filter. The collected 3D movies were further deconvolved applying the Huygens deconvolution software (SVI) and 3D volumes reconstructed using the MIP-rendering method from SVI (Netherlands). TVN vesicles were tracked and a maximum projection generated using the Manual Tracking plugin form FIJI.

All movies were processed and assembled using FIJI (URL: http://fiji.sc/Fiji)^52^.

### Liver Intravital Microscopy

Transgenic *gfp-lc3* mice were infected intravenously with approximately 1 × 10^6^ *Pb*mCherry or *P. berghei-*EXP1-mCherry sporozoites. Mice were anaesthetized with a mixture of 125 mg/kg ketamine (Ketasol, Graeub) 12.5 mg/kg xylazine (Xylasol, Graeub) at the indicated time post-infection, continually monitoring their vital function. The microsurgery was performed as previously described^53^. Intravital microscopy was conducted on an inverted Axiovert 200 M from Zeiss with a LSM 5 live scanning module and a temperature-controlled environmental chamber. The exposed liver lobe was imaged using a Plan Apochromat 63×/1.4 NA DIC M27 oil-immersion objective from Zeiss. GFP was excited by applying 5 mW of 489 nm light from a diode laser and the emission collected through a 495-555 band pass filter, while 2 mW from a 561 nm diode pumped solid-state (DPSS) laser and the BP 575-615 + LP 655 emission filter was used for the detection of mCherry. Dual fluorescence was acquired as single tracks. LC3 coverage around the *P. berghei* parasite was quantified using the software FIJI, measuring the contours of the parasite (defined as 100%), and the coverage by LC3 (expressed as a percentage of the total contour). Quantifications of vesicle distribution were performed using FIJI to define the contours of the cell, the length between the parasite and the host cell surface, and the number of vesicles. Based on these measurements, the total length was divided into 3 sections. Vesicles within the first third of the total length between the parasite and the host surface were classified as “proximal”; vesicles in the middle third division were classified as “intermediate”, and vesicles in the furthest division (closest to the host cell surface) were classified as “distal”. Quantifications are displayed as percentage of total. All quantifications were based on 10-20 intravital images from 5-6 mice per time point. The recorded movies were assembled and processed using the FIJI software (http://fiji.sc/Fiji)^52^.

### Photobleaching Experiments

Infections were performed as described for live cell imaging and bleaching experiments conducted on the Andromedar spinning disc with Yanus laser scan head from Till Photonics using the 40 × 1.35 NA oil-immersion objective from Olympus. To ensure stable environmental conditions the Ludin Chamber (Life Imaging Services, Switzerland) was equilibrated to 37 °C and 5% CO_2_. After acquiring five time points, bleaching was performed with 80% power of a 488 solid-state laser in a diffraction-limited ROI and a total dwell time of 3 ms. Fluorescence recovery of the GFP signal was recorded every 0.5 s over a period of 3 min using a 525/50 (BrightLineHC) filter. To ensure fast acquisition speed during fluorescence recovery, mCherry images of the parasite were only obtained during the pre-bleach duration. Prior to intensity quantifications for FRAP, parasite drift was corrected (StackReg Plugin, Fiji). Mean intensity was measured in the bleach region with an ROI diameter of 2.5 pixel and pixel size corresponding to 0.15 µm. Intensities were measured using FIJI software. Fluorescence intensity was normalized to the pre-bleached images and averaged intensity fitted by double normalization.

FLIP experiments were performed under equivalent experimental settings as described above. Adaptations comprise imaging interval of 2.4 s and the total duration of 5 min. Additionally to the GFP, also mCherry was acquired continuously using the 561 nm solid-state laser and 594 LP Edge Basic long-pass filter. Parasites that had drifted out of the bleach region during acquisition were excluded from the subsequent quantification.

### Photoconversion Experiments

For all photoconversion experiments infections were performed as described for live cell imaging. Quantitative photoconversion experiments were conducted on the Andromedar spinning disc with Yanus laser scan head from Till Photonics and under stable environmental conditions (37 °C and 5% CO_2_). Time-lapse acquisition was performed with the 60×/1.35 NA oil-immersion objective from Olympus and the Andor 897 high-speed EMCCD camera with an additional 1.2x magnification lens in front. After acquiring five time points, photoactivation was performed with 5% power of a 405 nm laser diode in a diffraction-limited ROI. Fluorescence was captured every 0.5 s for 1.5 min, excited with a 488 nm and 561 nm wavelengths and emission collected through a multi band dichroics 405/490/561/640 nm filter. Drifted parasites were corrected using the StackReg plugin (FIJI). Pixel intensity changes were measured in a 3 × 3 pixel rectangular area within the activation ROI (1 pixel = 0.1 µm) using multi kymograph plugin (FIJI). Fluorescence intensity was normalized to the first post-activation image and averaged intensity fitted by double normalization.

Time-lapse microscopy of the photoactivated Dendra2 was conducted on the previously described inverted Leica TCS SP8 at a temperature of 37 °C and 5% CO_2_ environment using 63×/1.4 NA oil objective. Dendra2 was activated using the 405 nm Diode laser at 2%. Subsequent imaging was performed﻿ by sequential excitation at 490 nm and 553 nm and an absorption range between 500–535 nm and 560–705 nm using the photomultiplier tube (PMT) detectors.

The PAGFP-ubiquitin time-lapse was recorded on the Andromedar spinning disc microscope using settings described for the quantitative photoconversion experiment. Photoactivation was acquired with 5% 405 nm power and the first images captured with 1 s/frame over 0.5 min and subsequently with an interval time of 2 min for an additional 40 cycles using 488 nm excitation wavelength for PAGFP-Ub and 561 nm pmRFP-LC3 and the emission multi band dichroics 405/490/561/640 nm.

### Statistics

Graphical display of the data was generated with Prism 7. Error bars represent standard deviation calculated from at least 3 independent experiments. For the data plotted in a box plot *P*-values were determined by applying one- or two-way ANOVA, respectively. No data were excluded form quantification unless indicated. No statistical method was applied to determine sample size.

## Electronic supplementary material

Supplemental Information

Movie S1. Dynamics of the LC3-positive TVN in vivo.

Movie S2. LC3 concentrates in the TVN in vivo.

Movie S3. Loss of vacuole-associated LC3 in mature liver schizonts in vivo.

Movie S4. Fate of the parasite-associated LC3 followed over the P. berghei liver stage development in vitro.

Movie S5. TVN expansion contributes to LC3 accumulation.

Movie S6. TVN dynamics contribute to the spatial control of GFP-LC3.

Movie S7. The early stage PVM-protein UIS4 confirms LC3-association with the TVN.

Movie S8. Clustering of LC3 at the PVM of young liver schizonts.

Movie S9. The TVN of mature schizonts becomes rather stationary and stunted.

Movie S10. Compartment-specific mobility of parasite-associated GFP-LC3.

Movie S11. Trapping of GFP-LC3 in the TVN.

Movie S12. Spatial control of LC3 begins with TVN-evolvement of sporozoites.

Movie S13. Immediate response of sporozoites on LC3 incorporation into the PVM.

Movie S14: Ubiquitin and LC3 are simultaneously disposed into the TVN.

Movie S15. Vesicular shedding of LC3 from the TVN.

Movie S16. Tracking of EXP1-vesicles by 4D imaging.

Movie S17: Vesicular shedding of Dendra2-LC3.
