# Supplementary material for: Shedding of host autophagic proteins from the parasitophorous vacuolar membrane of Plasmodium berghei
Source: Sci Rep. 2017 May 19;7:2191. doi: 10.1038/s41598-017-02156-7 (PMC5438358; doi:10.1038/s41598-017-02156-7)
Supplement: Supplementary file 1 — Supplemental Information [file 41598_2017_2156_MOESM1_ESM.pdf]

Supplemental Information

Shedding of host autophagic proteins from the parasitophorous vacuolar membrane of *Plasmodium berghei*

Carolina Agop-Nersesian, Mariana De Niz, Livia Niklaus, Monica Prado, Nina Eickel, Volker T. Heussler

Figure S1

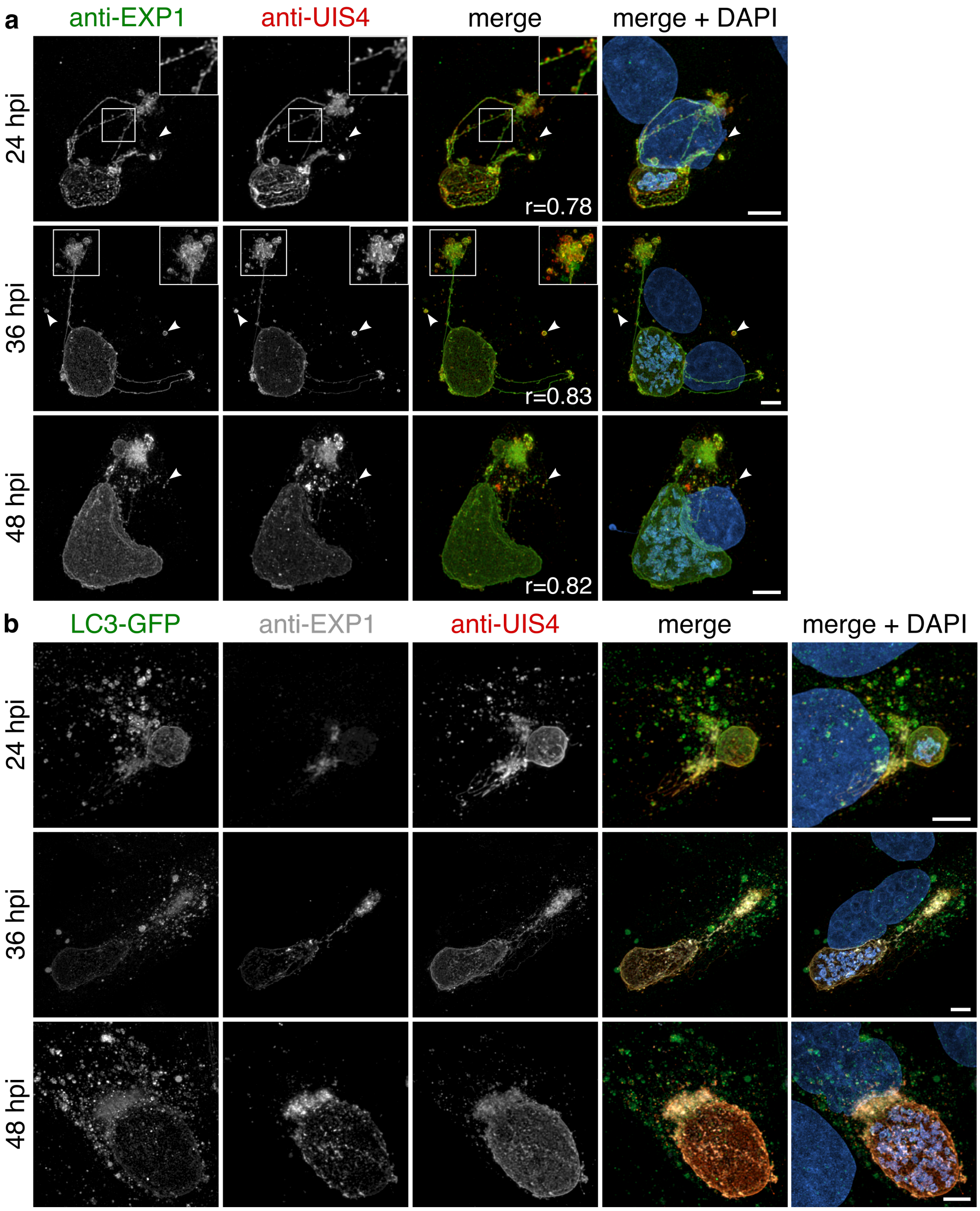

a
anti-EXP1
anti-UIS4
merge
merge + DAPI
24 hpi
36 hpi
48 hpi
r=0.78
r=0.83
r=0.82
b
LC3-GFP
anti-EXP1
anti-UIS4
merge
merge + DAPI
24 hpi
36 hpi
48 hpi

Figure S2

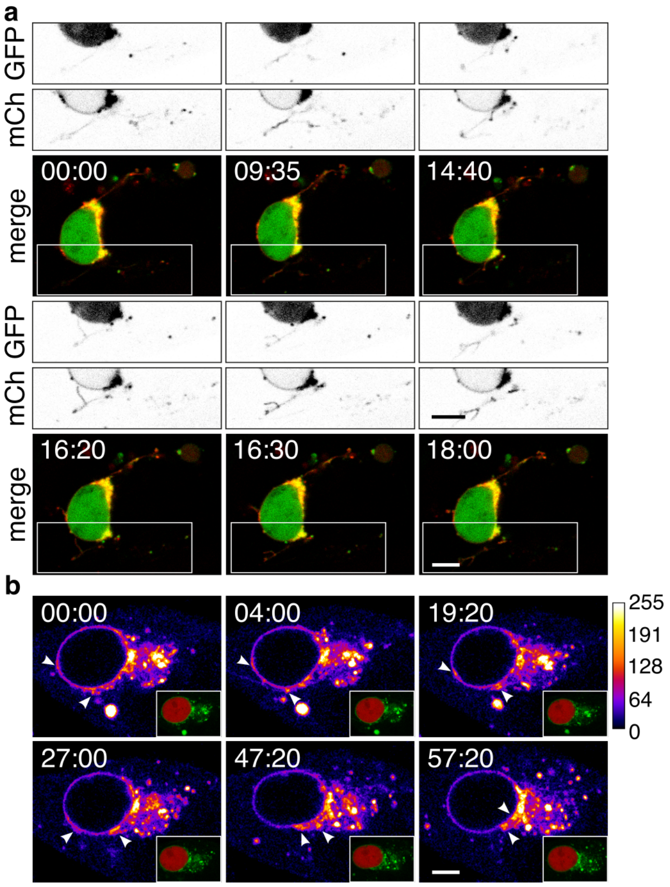

a
GFP
mCh
merge
00:00
09:35
14:40
GFP
mCh
merge
16:20
16:30
18:00
b
00:00
04:00
19:20
27:00
47:20
57:20
255
191
128
64
0

Figure S3

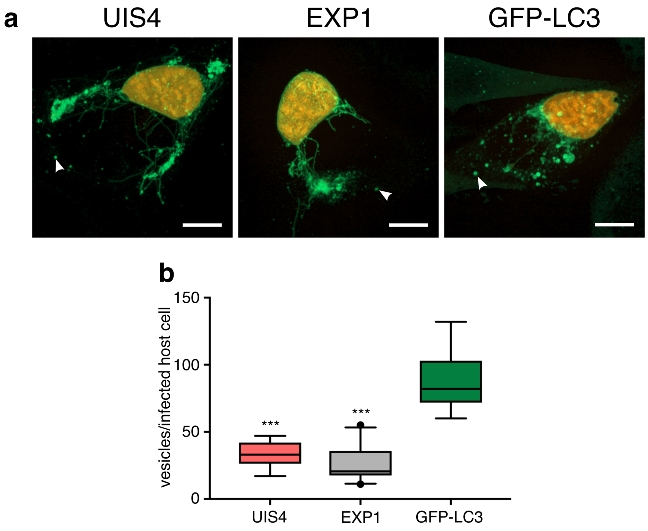
a
UIS4
EXP1
GFP-LC3
b
vesicles/infected host cell
150
100
50
0
***
***
UIS4
EXP1
GFP-LC3

Figure S1. Various PVM-localized proteins share common areas in the TVN. (a) 3D maximum intensity projection (3D-MIP) of liver schizonts co-stained for the PVM proteins anti-EXP1 (green) and anti-UIS4 (red) at the indicated time points. The TVN is composed of numerous branched tubular structures, several node-like clusters and PVM-derived vesicles (white arrowhead). The co-localization correlation is determined by the Pearson coefficient (r). **(b)** 3D-MIP of wild type parasites in GFP-LC3 (green) expressing cells. Parasites were fixed at the indicated time points and the PVM labelled against anti-EXP1 (grey) and anti-UIS4 (red). Nuclei are visualized with the DNA dye DAPI. Scale bars 5 µm for the 24 h time point and 10 µm for later stages.

Figure S2. Membrane-bound LC3 accumulates in the dynamic TVN. (a) Time-lapse movie of a *P. berghei*-infected GFP-LC3 expressing HeLa cell 24 hpi. Parasites are co-expressing *Pb*UIS4-mCherry and cytoplasmic GFP. **(b)** Time-lapse movie of *Pb*mCherry-infected HeLa cells stably expressing GFP-LC3 30 hpi. The GFP-LC3 surrounding a young liver schizont is displayed in pseudo-colours as correlation to the respective intensity values. Inset shows the merge image of a liver schizont (red) surrounded by GFP-LC3 (green). LC3 accumulates in membrane patches at the PVM, which move towards the TVN (white arrowheads). Colour calibration bar on the right. Imaging interval 20 s. Time stamp, min:s. Scale bar, 5 µm.

Figure S3. Host-autophagy machinery remains active in host cells infected with young liver schizonts. (a) Representative 3D-MIP of young schizonts (*Pb*mCherry, 36 hpi). Images were obtained by spinning disc microscopy with

0.15 µm z-increments. The PVM-derived vesicles were visualized either with anti-UIS4 or anti-EXP1. LC3-vesicles were determined in GFP-LC3 infected cells. Scale bars, 10 µm. **(b)** Total number of PVM-derived and LC3 vesicles per infected host cell. (n=15, ***P<0.001, one-way ANOVA).

Movie S1. Dynamics of the LC3-positive TVN *in vivo*. Intravital microscopy on the liver of *gfp-lc3* transgenic mice infected with a young liver schizont (24 hpi) was imaged with 2 min intervals. The PVM of *Pb*mCherry schizonts (red) is recognized by host GFP-LC3 (green). White arrowhead indicates the formation of a LC3-positive protrusion extending into the hepatocyte cytoplasm. Time stamp, min:s. Scale bar, 10 µm.

Movie S2. LC3 concentrates in the TVN *in vivo.* Intravital microscopy on the liver of *gfp-lc3* transgenic mice infected with a proliferating liver schizont (30 hpi) was imaged with 5 s intervals. The *Pb*mCherry liver schizont (red) contains a LC3-positive TVN (green). White arrowhead follows the evolvement of the fine-branched network into multiple robust tubules. Associated with these tubules is a constant increase of GFP-LC3 intensity. Time stamp, min:s. Scale bar, 10 µm.

Movie S3. Loss of vacuole-associated LC3 in mature liver schizonts *in vivo.* Intravital microscopy on the liver of *gfp-lc3* transgenic mice infected with a proliferating liver schizont (44 hpi) was imaged with 2 s intervals. *Pb*mCherry parasites (red), which have successfully developed into mature liver schizonts, remove host LC3 (green) from their vacuolar surface. LC3-GFP can be found exclusively in the hepatocyte cytoplasm. Time scale, min:s. Scale bar, 10 µm.

Movie S4. Fate of the parasite-associated LC3 followed over the *P. berghei* liver stage development *in vitro*. Long-term wide field microscopy of GFP-LC3 HeLa cells (green) infected with *Pb*mCherry (red), imaged every 30 min. Invaded sporozoites, differentiating and proliferating successfully into mature liver schizont clear LC3 from the vacuolar surface. Time stamp, h:min. Scale bar, 10 µm.

Movie S5. TVN expansion contributes to LC3 accumulation. *In vitro* time-lapse microscopy of 33 hpi young EXP1-mCherry liver schizont in a GFP-LC3 HeLa cell, imaged in 5 min intervals. EXP1-mCherry (red) driven by the late liver stage promoter, LISP2. Relocation of the exported EXP1-mCherry into the existing LC3-positive TVN starts to become prominent from 30 h onwards. With expansion of the TVN (white arrowhead), LC3 is progressively removed form the PVM (red arrowhead). Time stamp, h:min. Scale bar, 10 μm.

Movie S6. TVN dynamics contribute to the spatial control of GFP-LC3. *In vitro* time-lapse microscopy of 30 hpi EXP1-mCherry young liver schizonts infecting GFP-LC3 HeLa cells imaged every 30 s. The TVN composed of non-motile cisternae-like clusters, fast moving tubular structures (white arrowhead) and PVM-derived vesicles (red arrowhead) containing GFP-LC3. LC3 accumulates especially in the large clusters. Time stamp, min:s. Scale bar, 5 μm.

Movie S7. The early stage PVM-protein UIS4 confirms LC3-association with the TVN. Fast-iterative *in vitro* imaging of GFP-LC3 HeLa cells infected with *Pb*UIS4-mCherry 24 h liver schizonts, co-expressing cytoplasmic GFP. GFP-LC3 (green) accumulates in the TVN-clusters (red). UIS4-positive TVN contains analogous features and dynamics as determined by EXP1 and GFP-LC3. Acquisition interval 5 s. Time stamp, min:s. Scale bar, 5 μm.

Movie S8. Clustering of LC3 at the PVM of young liver schizonts. Fast-iterative *in vitro* imaging of a GFP-LC3 HeLa cell infected with a *Pb*mCherry 24 h liver schizont. Parasite-associated GFP-LC3 shown in pseudo-colours accumulates in LC3-patches (white arrowheads) moving into the convoluted TVN. The respective

merged image of the red fluorescing schizont and GFP-LC3 in green is shown in the inset. Acquisition interval 20 s. Time stamp, min:s. Scale bar, 5 µm.

Movie S9. The TVN of mature schizonts becomes rather stationary and stunted. Intravital microscopy on the liver of *gfp-lc3* transgenic mice infected with mature EXP1-mCherry liver schizonts 41 hpi, imaged with 5 s intervals. Residual LC3 (green) becomes restricted in the TVN (red). With completion of the replicative phase, the TVN becomes rather stationary and stunted. Time scale, min:s. Scale bar, 10 µm.

Movie S10. Compartment-specific mobility of parasite-associated GFP-LC3. Fluorescence Recovery After Photobleaching (FRAP) microscopy of GFP-LC3 HeLa cells infected with young liver schizonts. *Pb*mCherry schizonts were bleached either at the PVM (orange ROI), left panel or at the TVN (green ROI), right panel. Inset shows merge of the parasite in red and LC3 in green. PVM-associated LC3 recovers significantly faster than at the TVN. Time stamp, s. Scale bar, 10 µm.

Movie S11. Trapping of GFP-LC3 in the TVN. Fluorescence Loss In Photobleaching (FLIP) microscopy on GFP-LC3 HeLa cells infected with young liver schizonts. Left panel: repetitive bleaching of neighbouring host cells as control for imaging-induced fluorescent decay. Right panel: repetitive bleaching of the PVM. PVM-associated LC3 is progressively bleached, while the fluorescent signal at the TVN is largely retained. Time stamp, s. Scale bar, 10 µm.

Movie S12. Spatial control of LC3 begins with TVN-evolvement of sporozoites. Fast-iterative wide field microscopy of *Pb*mCherry sporozoites in GFP-LC3 HeLa cells (1 hpi). The PVM outlining the sporozoite and the fine-branched tubular network is visualized by GFP-LC3. The TVN dynamics were imaged in 30 s intervals.

Arrowhead indicates the increasing accumulation of LC3 in the nascent cisternae-like cluster of the TVN. Time stamp, min:s. Scale bar, 5 µm.

Movie S13. Immediate response of sporozoites on LC3 incorporation into the PVM. Photoconversion of Dendra2-LC3 at the tip of wild type sporozoites with developing TVN. Left panel: Photoconverted Dendra2-LC3 in the red channel. Right panel: Non-activated Dendra2-LC3 in the green channel. Activated Dendra2-LC3 accumulates in membrane patches, finally exiting into the nascent TVN (black arrowhead). Time stamp, min:s. Scale bar, 5 µm.

Movie S14: Ubiquitin and LC3 are simultaneously disposed into the TVN. Photoactivation of PAGFP-UB in HeLa cells co-transfected with pmRFP-LC3 at a ROI opposing the sporozoite's TVN. Left panel: PAGFP-UB. Right panel: pmRFP-LC3. The autophagic complex concentrates in patches and moves into the posteriorly located TVN of the sporozoite (black arrowhead). Time stamp, min:s. Scale bar, 5 µm.

Movie S15. Vesicular shedding of LC3 from the TVN. 3D movie of 24 h LC3-positive liver schizonts recorded with 0.25 µm increments and 30 s intervals. The LC3-positive TVN features rather stationary cisternae-like clusters and highly motile tubular protrusions. The second half of the movie shows the magnification of the TVN. White arrowhead indicates the budding event of a LC3-positive vesicle. Red arrowhead follows the LC3-positive TVN protrusion. Time stamp, min:s. Scale bar, 10 µm.

Movie S16. Tracking of EXP1-vesicles by 4D imaging. 3D movie of EXP1-mCherry expressing liver schizonts (33 hpi) recorded with 0.4 µm increments and 15 s intervals. White arrowhead highlights a TVN tubular protrusion. Subsequent

movie repetition shows the movement of EXP1-vesicles tracked through the series of image volumes. Time stamp, min:s. Scale bar, 10 μm.

Movie S17: Vesicular shedding of Dendra2-LC3. Photoconversion of Dendra2-LC3 at the PVM of 33 hpi young *Pb*GFPcon schizonts. Activated red-shifted Dendra2-LC3 shown on the left panel and non-activated Dendra2-LC3 and the cytoplasmic GFP of the liver schizonts on the right. Black ROI indicates area of photoconversion. Activated LC3, imaged every 2 min, moves from ROI (arrowhead outline) and accumulates at the TVN junction, where it finally becomes shed as a vesicle (black arrowhead) into the host cytoplasm. Time stamp, min:s. Scale bar, 5 μm.
